# The burden of recording and reporting health data in primary health care facilities in five low- and lower-middle income countries

**DOI:** 10.1186/s12913-021-06652-5

**Published:** 2021-09-13

**Authors:** Amani Siyam, Por Ir, Dararith York, James Antwi, Freddie Amponsah, Ofelia Rambique, Carlos Funzamo, Aderemi Azeez, Leonard Mboera, Claud John Kumalija, Susan Fred Rumisha, Irene Mremi, Ties Boerma, Kathryn O’Neill

**Affiliations:** 1 Health Workforce Department, World Health Organization, Avenue Appia 20, CH-1211 Geneva, Switzerland; 2 National Institute of Public Health, No. 80, Samdach Penn Nouth Blvd (289), Sangkat Boeungkak 2, Tuol Kork District, Phnom Penh, Cambodia; 3 Department of Planning and Health Information, Ministry of Health, No. 80, Samdach Penn Nouth Blvd (289), Sangkat Boeungkak 2, Tuol Kork District, Phnom Penh, Cambodia; 4 Centre for Health and Social Policy Research, West End University College, Ngleshie Amanfro, Accra, Ghana; 5 Ghana Health Service, Private Mail Bag, Ministries, Accra, Ghana; 6 National Institute of Health, Vila de Marracuene, National Road, 3943 Maputo, Mozambique; 7 World Health Organization Country Office, Rua Joseph Ki-zerbo 227, P.O. Box 377, Maputo, Mozambique; 8 Federal Ministry of Health, Federal Secretariat, Phase III, Shehu Shagari Way, Central Business District, Abuja, FCT Nigeria; 9 SACIDS Foundation for One Health (SACIDS), Sokoine University of Agriculture (SUA), P.O. Box 3297, Chuo Kikuu, SUA, Morogoro, Tanzania; 10 Health Management Information System (HMIS), Ministry of Health, Community Development, Gender, Elderly and Children, Dodoma, Tanzania; 11 The National Institute for Medical Research, 3 Barack Obama Drive, P.O.Box 9653, 11101 Dar es Salaam, Tanzania; 12 Department of Community Health Sciences, Max Rady College of Medicine-University of Manitoba, Room S113 - 750 Bannatyne Ave, Winnipeg, MB R3E 0W3 Canada; 13 Integrated Health Services Department, World Health Organization, Avenue Appia 20, CH-1211 Geneva 27, Switzerland

**Keywords:** Health worker, Time motion, Public PHC facilities, Registers, Reporting forms

## Abstract

**Background:**

Recording and reporting health data in facilities is the backbone of routine health information systems which provide data collected by health facility workers during service provision. Data is firstly collected in a register, to record patient health data and care process, and tallied into nationally designed reporting forms. While there is anecdotal evidence of large numbers of registers and reporting forms for primary health care (PHC) facilities, there are few systematic studies to document this potential burden on health workers. This multi-country study aimed to document the numbers of registers and reporting forms use at the PHC level and to estimate the time it requires for health workers to meet data demands.

**Methods:**

In Cambodia, Ghana, Mozambique, Nigeria and Tanzania, a desk review was conducted to document registers and reporting forms mandated at the PHC level. In each country, visits to 16 randomly selected public PHC facilities followed to assess the time spent on paper-based recording and reporting. Information was collected through self-reports of estimated time use by health workers, and observation of 1360 provider-patient interactions. Data was primarily collected in outpatient care (OPD), antenatal care (ANC), immunization (EPI), family planning (FP), HIV and Tuberculosis (TB) services.

**Result:**

Cross-countries, the average number of registers was 34 (ranging between 16 and 48). Of those, 77% were verified in use and each register line had at least 20 cells to be completed per patient. The mean time spent on recording was about one-third the total consultation time for OPD, FP, ANC and EPI services combined. Cross-countries, the average number of monthly reporting forms was 35 (ranging between 19 and 52) of which 78% were verified in use. The estimated time to complete monthly reporting forms was 9 h (ranging between 4 to 15 h) per month per health worker.

**Conclusions:**

PHC facilities are mandated to use many registers and reporting forms pausing a considerable burden to health workers. Service delivery systems are expected to vary, however an imperative need remains to invest in international standards of facility-based registers and reporting forms, to ensure regular, comparable, quality-driven facility data collection and use.

**Supplementary Information:**

The online version contains supplementary material available at 10.1186/s12913-021-06652-5.

## Background

A health information system has been defined as a collective effort to capture, process, report, and use health information at each level of the health system [1]. Regular data collected and reported by health facilities, often referred to as the routine health information system (RHIS) or health management information system (HMIS), is a core component of country systems [2, 3]. Such routine data are collected by health workers during service provision, describing an event, procedure, or resources associated with a given health institution.

Routine health facility data are collected to inform patient and program management, and to generate health statistics on, for instance, the coverage of immunization or antenatal care for a district or country. Even though health workers spend considerable time on recording and reporting of data, only few studies have examined the actual burden to health care professionals in PHC settings in low-income (LIC) and lower middle-income (LMIC) countries. Current evidence varies between settings, types of health care workers, and types of services such as community outreach versus in-facility promotion, preventive and curative services [4–9]. Averagely, in a working shift of 6–7 h, the time motion of health workers divides into 50–60% of productive direct patient care and the remaining proportion spread between a range of supporting activities, including recording and reporting [5–7].

The recording of patient care generally takes place during the consultation period. This is commonly done using a register which is a document used to record patient data ranging from a registration number, name, age, sex and health data pertinent to the patient care process. There is no standard for the optimal consultation time per service area or time allocated to recording [7]. Yet, health workers are expected to provide quality care and allocate adequate time to accurately record the details of the care provided, whether this is paper-based or electronic. Recording of information includes line-listing of care, completion of patient cards (that may or may not be kept in facilities) and tally sheets (for services such as immunisation and out-patient care [10–12]). In most LICs and LMICs, recording in PHC facilities is predominantly paper-based.

Reporting is also a fundamental requirement of a national RHIS [13]. Commonly, data collected by registers data are collated and tallied onto nationally designed standard reporting forms. This includes reporting data on service utilization, health status at clinical encounters, vital events, interventions delivered, outcomes of interventions, and health services resources. The frequency of reporting varies according to the data type, information needs and system capacity, but is most commonly monthly for routine service data. The reports are usually communicated to a district health office (either electronically, by hand, or collected as part of supervisory visits). In many rural settings, it is at this level that the data is compiled and entered into an electronic system for analysis and transmission to the national level.

With increased demand for more health indicators and more disaggregated data, public health experts and clinicians have expressed concerns about the amount of time health workers spend on recording and reporting data, at the potential cost and risk to quality service provision. In this multi-country study, we aimed to document the numbers of registers and reporting forms in use and estimate the time it requires for health workers to meet these demands for data.

## Methods

Five LIC and LMICs were invited to participate in the study: Cambodia, Ghana, Mozambique, Nigeria and Tanzania. The countries were selected based on an expression of interest by the Ministry of Health (MOH) during WHO capacity-building workshops on RHISs. Each country study was led by the MOH and a principal investigator from a country public health institute. A national desk review was conducted to collect information on all recording registers and reporting forms mandated for use at the PHC level. A register refers to a document used to record patient data ranging from a registration number, name, age, sex and health data pertinent to the patient care process. Registers data are subsequently collated and tallied onto nationally designed standard reporting forms. Those are reviewed by the head of the facility before submission, mostly on monthly basis, to the district management office (DMO). Reporting forms data are then entered into the district management information system (DMIS) for further analysis and use at all levels of the health system.

Based on a common protocol, each country was recommended to randomly select four districts and four public PHC facilities within each district, i.e. 16 in total, for facility visits and district office interviews. The fieldwork, preceded by pilot-testing, multi-country and country training workshops, was conducted during 2016 and 2017. The field teams consisted mainly of 3–5 individuals familiar with the collection and analysis of routine health facility data. For all registers and reporting forms, a standard set of variables was collected. For registers, the number of mandatory (and conditional) data cells to be filled for every patient was counted, and for reporting forms, the number of data cells, the mandatory ones, and those due to disaggregation were counted.

In each country setting, interviews and observations were conducted in two separate visits. The first visit was made to all 16 facilities to review and verify registers and reporting forms in use and to interview health workers who self-reported to the data collectors the estimated time use for recording and reporting. Information on the number of health workers by occupation, and, data on service-specific patient loads were collected for the three preceding months. The second visit involved the observation of recording practices (over a period of 2 weeks during peak and non-peak times) in five of the 16 health facilities for six frontline services: OPD, ANC, EPI, FP, HIV/AIDS and TB. In the case of routine laboratory requests as part of the diagnostic process, the field teams computed consultation and recording time as the sum of time spent during initial consultation and after visiting the laboratory.

The data collection, quality checks and analysis were carried out in Microsoft Excel and Stata 16.2. Times-based data was summarized as medians and means, the latter used for most of the analyses presented in the paper. Ethical approval for the study was obtained from the responsible authorities in each country setting. The multi-country study was exempted from WHO Ethical Committee Review as there was no possibility of harm arising as a result of the conduct of the research involved.

## Results

### Characteristics of the PHC facilities

The characteristics of the 80 public PHC facilities slightly varied between the five countries (Table 1). Most facilities were located in rural settings (62 out of 80), except in Mozambique, and provided only ambulatory care (63 of the 80 facilities). Almost all facilities (70 out of 80) provided OPD, FP, ANC, EPI and child well-care (Appendix Table 1a). The median number of health professionals (physicians, nursing and midwifery) varied considerably from less than three in Community-Based Health Planning and Services (CHPS) facilities in Ghana to 12 health professionals in Mozambique health centres (Appendix Table 1b). None of the facilities had designated health information staff. The patient loads also varied considerably between countries and by services. Overall, the daily total number of patients for the six services per health worker ranged from 10 in Nigeria and Tanzania to 33 in Mozambique (Table 1).

**Table 1:** Key characteristics of 80 Primary Health Care (PHC) facilities, five countries (2016–2017)

		Cambodia	Ghana	Mozambique	Nigeria	Tanzania
Number	Health facilities visited	16	16	16	16	16
Location	Urban	0	0	8	3	4
	Peri-urban	2	1	0	0	
	Rural	14	15	8	13	12
Type	Health Centre	16	5	15	12	4
	Health Post/ CHPS/ Health Dispensary	0	10	1	3	12
	Maternal/Child Health Clinic	0	1	0	1	0
Outpatient care only	No	2	3	5	3	4
	Yes	14	13	11	13	12
Number of health staff	Clinician / nurse / midwife/ AMO / CO	6	2	12	1	4
	CHW/CHN/CHEW	0	3	1	4	0
Workload	Patient load per month	1860	1041	8728	429	837
	Patient load per day	85	47	397	20	38
	Patient load per day per health staff	14	24	33	10	10

*CHPS* Community-Based Health Planning and Services, *AMO* Assistant Medical Officer, *CO* Clinical Officer, *CHW* Community Health Worker, *CHN* Community Health Nurse, *CHEW* Community Health Extension Worker

### Recording the PHC facility data

**Fig. 1:**
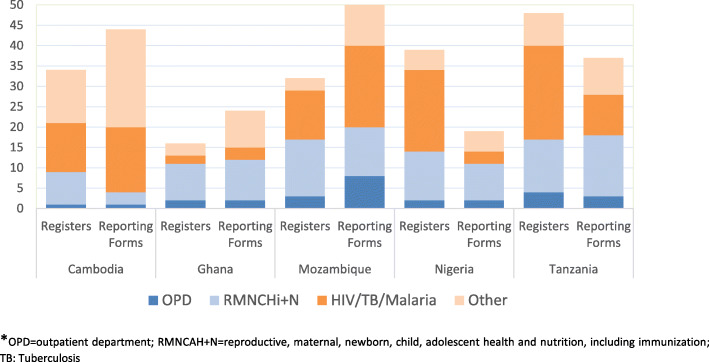
The number of registers and reporting forms mandated for use in PHC facilities by programme areas*, five countries (2016–2017)

**Fig. 2:**
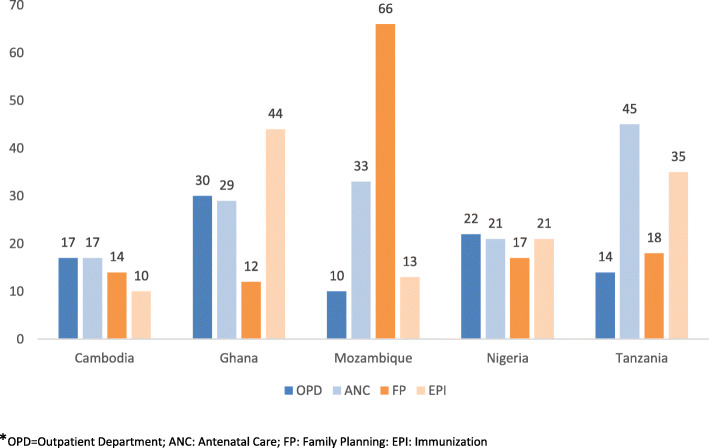
Number of mandatory cells in PHC facility registers* for OPD, ANC, FP and EPI, five countries (2016–2017)

On average, 34 registers were identified through the national desk review (Fig. 1) ranging from 16 registers in Ghana to 48 in Tanzania. Most registers are for reproductive, maternal, newborn, child and adolescent health (RMNCAH) and nutrition services with 8–14 registers per country. Nigeria and Tanzania had as many as 15 and 22 registers for HIV, respectively. All registers originated from the MOH and most introduced in the years 2013/2014. During the first facility visit, 77% of the inventory-identified registers were verified to be in use (Appendix Table 2). The mandatory number of fields or cells to be completed for each patient was assessed in four high volume registers and varied considerably between countries and services (Fig. 2). On average, a register required 25 cells to be completed for a patient, ranging from an average of 19 cells for OPD services to 29 cells for ANC services (Appendix Table 3a).

**Fig. 3:**
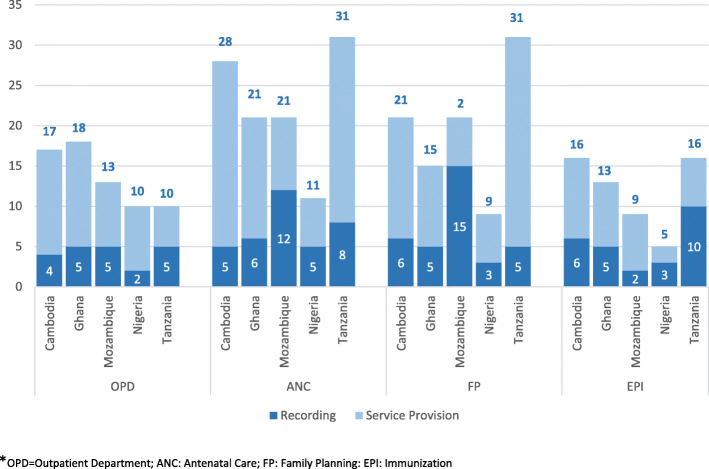
The mean Consultation time (service provision and recording) in minutes for OPD, ANC, FP and EPI services, five countries (2016–2017)

Figure 3 compares the mean time in minutes spent on a consultation and on recording across four service areas, based on health workers interviews (self-reports). For OPD visits, the estimated mean consultation time ranged from 10 min in Nigeria and Tanzania to 18 min in Ghana. The time to fill the register was 2–5 min (24–50% of the consultation time) in four countries. Only in Nigeria PHC facilities, where service delivery was mostly through community services (65% of the community nurses/workers service provision time), the consultation time was generally shorter and the time spent on recording was only 2 min. The greater the number of cells to be completed the longer the self-reported time to record the information in the register (about 1 min for six cells).

The proportion of the consultation time spent on recording was fairly consistent across countries and services (Appendix Table 3a). On average, the percentage of time spent on recording was 31% of an OPD consultation, 32% of an ANC consultation, 35% of a FP consultation and 44% of an EPI consultation. Overall, the mean percentage of recording time across the four services was 26% for Cambodia, 31% for Ghana, 32% for Tanzania, 37% for Nigeria, and 53% for Mozambique.

HIV and TB services showed wider variability in the number of registers and the time for consultation and recording. For instance, in Cambodia the HIV voluntary testing laboratory register had 768 cells but it reportedly took only 5 min out of an 18-min consultation to complete the register. The national HIV care and treatment register in Tanzania had 22 cells but took a reported 11 min out of a 40-min consultation (Appendix Table 3b).

Country teams observed a total of 1360 consultations (Appendix Table 4a) distributing as 31% OPD, 19% ANC, 17% FP, 21% EPI, 8% HIV/AIDS, and 3% TB. The comparison of the mean observed, and self-reported times showed that there is a tendency to overestimate the time spent on consultation and recording (Appendix Table 4b). The overall mean observed consultation time for the four high volume services - OPD, ANC, FP and EPI - was 10.5 min, compared 16.8 min self-reported time, an overestimate of 60%. The corresponding estimates for recording time were 5.9 min and 3.6 min, an overestimate of 63%. However, the proportion of consultation time spent on recording remained about the same: on average 33% for OPD services, 33% for ANC, 30% for FP and 47% for EPI. HIV/AIDS and TB consultations were less often occurring and fewer to observe (Appendix Table 4a) hence inadequate to compare between countries in the same vein as the forging four high volume services (for example no HIV consultations were observed in Cambodia and Ghana; in the case of TB, 1 consultation was possible to observe in Cambodia and none in Ghana and Tanzania).

### Reporting the PHC facility data

The national desk review identified on average 35 monthly reporting forms, ranging from 19 in Nigeria to 52 in Mozambique (Appendix Table 5). The highest number of reporting forms for a specific programme were indicated in Mozambique (12 for TB), Cambodia (11 for Malaria) and Tanzania (10 for HIV). Overall, 78% of the national inventory reporting forms were verified to be in use during the first facility visit (Appendix Table 5).

The self-reported time required to complete all monthly reporting forms (Table 2) varied from 10 h in Nigeria (with 13 reporting forms) to 65 h in Tanzania (with 29 reporting forms). The time per health worker was just 4 and 5 h in Mozambique and Nigeria, but 13 and 15 h in Tanzania and Ghana, respectively. The monthly reporting time per 1000 patients was 23 to 28 h for Cambodia, Ghana and Nigeria, however Mozambique (6 h) and Tanzania (78 h) were exceptions (Table 2). The distribution of reporting time also differed greatly by service area (Table 3, Appendix Table 6), with OPD services accounting for 48% of the reporting time in Cambodia, RMNCAH services for 34% in Ghana, EPI for 25% in Mozambique, and HIV services leading in Tanzania (22%) and Nigeria (31%). These differences can partly be explained by the number of cells in a form (a detailed description is available in Appendix Table 7). For example, in Cambodia, the main and only OPD reporting form (with 4632 data cells of which 732 cells are averagely filled-in) requires 21 working hours to complete each month as it is an integrated health facility reporting form incorporating multiple service areas and disease-related statistics. Nigeria PHC facilities used a main health facility reporting form for the RHIS (with 585 data cells) that requires an estimated 2 of the estimated 10 h total reporting time per month to be completed.

**Table 2:** The mean number of hours needed to complete monthly reporting forms by service area in 80 PHC facilities, five countries (2016–2017)

Key attribute	Cambodia	Ghana	Mozambique	Nigeria	Tanzania
Total time (hours) [A]	44	29	48	10	65
Patient load per month [B]	1860	1041	8728	429	837
Time per 1000 patients (hours) [A/B]	24	28	6	23	78
Time per health worker (hours)	7	15	4	5	13
No. of reporting forms in use	20	23	25	13	29

*OPD* Outpatient Department, *RMNCAH* Reproductive, Maternal, Newborn, Child, Adolescent health, *EPI* Immunization, *TB* Tuberculosis

**Table 3:** The percentage distribution of the time spent to complete monthly reporting forms (confirmed in use) by service area in 80 PHC facilities, five countries (2016–2017)

	Form(s)	(%)	Form(s)	(%)	Form(s)	(%)	Form(s)	(%)	Form(s)	(%)
OPD	1	(48)	2	(25)	3	(8)	1	(19)	3	(14)
MNCH	1	(1)	9	(34)	3	(17)	2	(6)	5	(16)
EPI	1	(2)	1	(4)	2	(25)	4	(11)	3	(6)
HIV	1	(1)	1	(2)	3	(12)	2	(31)	9	(22)
TB	1	(2)	1	(1)	7	(9)				
Malaria	5	(21)	1	(3)						
Other	10	(25)	8	(31)	7	(34)	4	(33)	9	(42)
Total	20	(100)	23	(100)	25	(100)	13	(100)	29	(100)
Total time (hours)		44		29		48		10		65

*OPD* Outpatient Department, *RMNCAH* Reproductive, Maternal, Newborn, Child, Adolescent health, *EPI* Immunization, *TB* Tuberculosis

## Discussion

Our study is likely one of the few done to systematically document the burden of recording and reporting PHC facilities health data in multiple settings. The desk review of registers showed that all countries used dozens of registers at the PHC level, with an average of 34 registers per country. Almost all current versions of the registers were introduced from 2013/2014, possibly as a result of higher demand for national operational data and global monitoring requirements of PHC outcomes. The bulk of the registers belong to RMNCAH and nutrition, HIV, TB and malaria programmes. Registers generally had more than 20 mandatory cells per patient, although there were major variations between services and countries. Given these variations, an overall estimate has limitations, and indicates approximately that recording takes up to one-third of the consultation time.

Similarly, the desk review indicated a sizeable number of mandated reporting forms at the PHC level, on average 35 per country, of which three quarters were verified in use. Consequent to registers, these forms were predominantly associated with RMNCAH and infectious disease control services. The self-reported time required to complete reporting forms varied widely but for each health worker took up at least half a day per month and in two countries about two working days. The most common practice across countries and facilities, was that patients’ data are entered daily in different registers and tally-sheets (in the case of Cambodia, Mozambique and Tanzania), and were then summarised (usually by 2–3 staff sitting together) in the last 2–3 days of the month to prepare the monthly reporting forms.

These findings corroborate concerns over the reporting burden for health workers in PHC facilities working in LICs and LMICs. The extent to which a significant number of recording and reporting forms is justifiable depends on what the data are used for. Some of data is used at the facility level for patient management and monitoring, district supervisory purposes or logistics such as ordering medicines and commodities. But most of data are collected to feed into the RHIS and produce statistics for programme monitoring.

Data quality is often a major limitation, hampering the use of facility data and derived health statistics [13–15]. The collection of large volumes of data through registers and reporting forms, often combined with limited training and supportive supervision as well as feedback, is likely to contribute to poor data quality [16, 17]. The need for greater rationalization of data collection and reporting as part of service provision has been acknowledged. For instance, a review of Nigeria’s National HMIS policy highlighted multiplicity/duplication, parallel reporting tools and platforms [18, 19]. Despite the conducted review and harmonization efforts of data collection carried-out in 2014, it was concluded that much more should be done to reduce the burden of data collection from health facilities in Nigeria. Similar challenges have been noted in the other countries, where efforts to simplify and streamline data recording and reporting are overpowered by demand for disaggregated data on an increasing number of health service areas. In particular, programmes that are heavily supported by donors tend to collect large volumes of data through health facilities.

There was a general tendency to overreport consultation and recording times. In several instance, there were explanations for the higher estimates from health workers. A consultation time tends to vary by patient volume and length of the queues of waiting patients, as well as by the extent to which the recall (or observation) concerned “a patient’s first visit”, which usually takes much longer time. Another key factor is the arrangement of services in each facility. In a clinic with few staff, the laboratory screening, blood pressure, and temperature measurement are provided by one person during a consultation, while in larger facilities another person may perform these functions prior to the consultation session.

### Limitations

We did not produce a combined overall estimate of the proportion of time that health workers spend on recording and reporting. The ways in which the PHC were organized and delivered affected the ability to collect comparable data on recording and reporting practices. Therefore, the generalizability of our findings is limited, given the large variation between countries which can only partly be explained by differences in their information systems.

Our design, relying on self-reports with observations from a third of the study’s PHC facilities, can only provide a crude assessment of the recording and reporting time. Time and motion studies would be able to provide much greater detail on the actual practices and burden of recording and reporting for health workers in PHC facilities. Time and motion studies tend to be labour-intensive and commonly done with small samples of participants who are shadowed for a specified period of time to record the time and activities observed [20–22]. Our study aimed to provide a general view of what issues should be considered in such studies including the selection of facilities to obtain generalizable results. We did not consider electronic data collection, as none of the selected health facilities had such systems in place.

## Conclusion

Public PHC facilities in the five countries under study were often required to use as many as 30 or more registers and reporting forms, both with a significant number of mandatory cells. Recording on registers may take as much as one third of consultation time and completing monthly reporting forms can take up to two working days per health worker. There is however considerable variability between countries, and type of services which limits the generalizability of these findings. The extent to which these data are necessary for the improvement of service provision and allocation of resources was not assessed in this study, but in general, the immediate uses of the data appear limited, at least for supportive supervision and the production of health statistics.

Our study demonstrates the need for further research on the usefulness of a multitude of registers and reporting forms at the PHC facility and the relevance and use of the data collected in improving service delivery. This should lead to greater efficiency and rationalization of data collection and reporting, which is likely to further improve data quality and greater use of data for decision-making at all levels of the health system.

There is great potential for a rapid shift from paper-based to electronic data collection which countries can achieve with reasonable pace and sustainable success [14, 23]. However, electronic systems do not necessarily alleviate the burden of recording [24]. Results from two systematic reviews show that electronic systems did not necessarily involve real-time data entry by health workers, rather that provider-patient consultations were still paper-based and later the health worker or clerk enters the information into the computer [25, 26].

Country health service delivery systems are expected to vary, however there remains an imperative need to invest in international standards of facility-based registers and reporting forms for PHC services, to ensure regular, comparable, quality-driven health data collection and use, more crucially in low-resource settings.

## Supplementary Information

**Additional file 1:Appendix Table 1a.** Number of facilities providing specific services in five countries (2016–2017). **Appendix Table 1b.** Facility attributes (Median staffing) in five countries (2016–2017). **Appendix Table 2.** The Desk Review national inventory of registers mandated and verified in use, 80 PHC facilities, five countries (2016–2017). **Appendix Table 3a.** High use registers (OPD, ANC, FP, EPI) – estimated consultation and recording time in five countries (2016–2017). **Appendix Table 3b.** Disease-specific registers– estimated consultation and recording time in five countries (2016–2017). **Appendix Table 4a** the number of consultations observed by service area in five countries (2016–2017). **Appendix Table 4b** Comparing the mean consultation and register completion time (observed and self-reported) in five countries (2016–2017). **Appendix Table 5 –** The Desk Review national inventory of reporting forms mandated and verified in use, 80 PHC facilities, five countries (2016–2017). **Appendix Table 6** The number of forms confirmed in use and the estimated reporting time (median) in monthly by service groupings in five countries (2016–2017). **Appendix Table 7 –** Distribution of reporting forms (cells and estimated time), by service area, in five countries (2016–2017).

## Data Availability

The data sets generated and/or analysed during the present study are available from the corresponding author on reasonable request.

## Abbreviations

ANC: Antenatal Care
DHIS: District Health Information System
DMO: District Management Office
EPI: Expanded Programme on Immunization
FP: Family Planning
HIV: Human Immunodeficiency Virus
HMIS: Health Management Information System
LIC: Low Income Countries
LMIC: Lower Middle Income Countries
MOH: Ministry Of Health
OPD: Out-patient care department
PHC: Primary Health Care
RHIS: Routine Health Information System
RMNCAH: Reproductive, Maternal, Newborn, Child and Adolescent health
TB: Tuberculosis
WHO: World Health Organization
