# Supplementary material for: The burden of recording and reporting health data in primary health care facilities in five low- and lower-middle income countries
Source: BMC Health Serv Res. 2021 Sep 13;21(Suppl 1):691. doi: 10.1186/s12913-021-06652-5 (PMC8436492; doi:10.1186/s12913-021-06652-5)
Supplement: Supplementary file 1 — Additional file 1:Appendix Table 1a. Number of facilities providing specific services in five countries (2016–2017). Appendix Table 1b. Facility attributes (Median staffing) in five countries (2016–2017). Appendix Table 2. The Desk Review national inventory of registers mandated and verified in use, 80 PHC facilities, five countries (2016–2017). Appendix Table 3a. High use registers (OPD, ANC, FP, EPI) – estimated consultation and recording time in five countries (2016–2017). Appendix Table 3b. Disease-specific registers– estimated consultation and recording time in five countries (2016–2017). Appendix Table 4a the number of consultations observed by service area in five countries (2016–2017). Appendix Table 4b Comparing the mean consultation and register completion time (observed and self-reported) in five countries (2016–2017). Appendix Table 5 – The Desk Review national inventory of reporting forms mandated and verified in use, 80 PHC facilities, five countries (2016–2017). Appendix Table 6 The number of forms confirmed in use and the estimated reporting time (median) in monthly by service groupings in five countries (2016–2017). Appendix Table 7 – Distribution of reporting forms (cells and estimated time), by service area, in five countries (2016–2017). [file 12913_2021_6652_MOESM1_ESM.docx]

**The burden of recording and reporting health data in primary health care facilities in five low- and lower-middle-income countries**

**Appendix Tables**

**Appendix Table 1a –Number of facilities providing specific services in five countries (2016-2017)**

| Service offered | Cambodia | Ghana | Mozambique | Nigeria | Tanzania |
| --- | --- | --- | --- | --- | --- |
| OPD | 16 | 16 | 16 | 16 | 16 |
| ANC | 16 | 14 | 16 | 16 | 16 |
| PMTCT (with ANC) | 16 | 12 | 5 | 8 | 16 |
| Obstetric and newborn care | 15 | 8 | 11 | 13 | 14 |
| Family planning | 16 | 15 | 15 | 16 | 14 |
| Immunization | 16 | 16 | 14 | 16 | 16 |
| Well Child Care | 16 | 16 | 15 | 9 | 16 |
| ART | 16 | 12 | 16 | 4 | 12 |
| HIV | 16 | 8 | 16 | 12 | 13 |
| TB | 16 | 7 | 13 | 6 | 6 |
| STI | 8 | 8 | 10 | 13 | 15 |
| NCD | 5 | 7 | 13 | 12 | 11 |
| Lab | 1 | 1 | 7 | 2 | 11 |

**Appendix Table 1b – Facility attributes (Median staffing) in five countries (2016-2017)**

| Staffing category | Cambodia | | Ghana | Mozambique | Nigeria | Tanzania |
| --- | --- | --- | --- | --- | --- | --- |
| All staff | 8 | | 5 | 19 | 10 | 6 |
| Health staff | 6 | | 5 | 16 | 5 | 5 |
| Medics / physicians | 0 | | 0 | 1 | 0 | 0 |
| Nursing | 2 | | 2 | 5 | 1 | 2 |
| Midwifery | 3 | | 0 | 5 | 0 | 0 |
| AMO/ Clinical officer | 1 | | 0 | 1 | 0 | 1 |
| Others | 0 | | 0 | 3 | 0 | 1 |
| CHW/CHN/CHEW/JCHEW | 0 | | 3 | 1 | 4 | 0 |
| % Absenteeism (22) | | NA | NA | 23.9 | 31.7 | 14.3 |

**Appendix Table 2. The Desk Review national inventory of registers mandated and verified in use, 80 PHC facilities, five countries (2016-2017)**

| **Registers Grouping** | **Cambodia** | | | **Ghana** | | | **Mozambique** | | | **Nigeria** | | | **Tanzania** | | |
| --- | --- | --- | --- | --- | --- | --- | --- | --- | --- | --- | --- | --- | --- | --- | --- |
|  | **Counted** | **Year In** | **In use** | **Counted** | **Year In** | **In use** | **Counted** | **Year In** | **In use** | **Counted** | **Year In** | **In use** | **Counted** | **Year In** | **In use** |
| OPD | 1 | 1 | 1 | 2 | -- | 2 | 3 | -- | 1 | 2 | 2 | 2 | 4 | 4 | 3 |
| RMNCAH | 6 | 3 | 4 | 7 | -- | 7 | 6 | -- | 6 | 7 | 3 | 7 | 7 | 4 | 7 |
| Immunization (EPI) | 1 | 1 | 1 | 2 | -- | 1 | 5 | -- | 3 | 5 | 5 | 4 | 2 | 1 | 2 |
| HIV_related | 2 | 2 | 2 | 1 | -- | 1 | 7 | -- | 5 | 15 | -- | 12 | 22 | 2 | 14 |
| Malaria_related | 6 |  | 6 |  | -- |  |  | -- |  | 3 | -- | 1 |  |  |  |
| TB_related | 4 | 4 | 4 | 1 | -- | 1 | 5 | -- | 4 | 2 | -- | 2 | 1 | 1 | 1 |
| Nutrition_related | 1 | 1 | 1 |  | -- |  | 3 | -- | 2 |  | -- |  | 4 | -- | 3 |
| Other | 13 | 5 | 11 | 3 | -- | 2 | 3 | -- | 1 | 5 | 2 | 3 | 8 | 4 | 4 |
|  | 34 | 17 | 30 | 16 |  | 14 | 32 |  | 22 | 39 | 12 | 31 | 48 | 16 | 34 |

**Year In – Year introduced** is 2013/2014 or later; Other=Includes a range of registers, e.g. finance, laboratory, NCD, pharma…etc; In Cambodia 11 registers year unknown; Mozambique, 12 registers are under-revision and 20 Unknown year; Nigeria 15 registers year unknown; Tanzania 25 registers year unknown

**Appendix Table 3a. High use registers (OPD, ANC, FP, EPI) – estimated consultation and recording time in five countries (2016-2017)**

|  |  | | | | | | |
| --- | --- | --- | --- | --- | --- | --- | --- |
|  | **OPD Register** | | | | | | |
|  | Average | **Median (mins)** | |  | **Mean (mins)** | |  |
|  | Cells | Consultation | Recording | % | Consultation | Recording | % |
| Cambodia | 17 | 15 | 3 | (20) | 17 | 4 | (24) |
| Ghana | 30 | 14 | 5 | (36) | 18 | 5 | (28) |
| Mozambique | 10 | 10 | 5 | (50) | 13 | 5 | (38) |
| Nigeria | 22 | 10 | 2 | (20) | 10 | 2 | (20) |
| Tanzania | 14 | 10 | 5 | (50) | 10 | 5 | (50) |
|  | **ANC Register** | | | | | | |
|  | Average | **Median (mins)** | |  | **Mean (mins)** | |  |
|  | Cells | Consultation | Recording | % | Consultation | Recording | % |
| Cambodia | 17 | 25 | 5 | (20) | 28 | 5 | (18) |
| Ghana | 29 | 20 | 3 | (15) | 21 | 6 | (29) |
| Mozambique | 33 | 20 | 10 | (50) | 21 | 12 | (57) |
| Nigeria | 21 | 10 | 3 | (30) | 11 | 5 | (45) |
| Tanzania | 45 | 30 | 5 | (17) | 31 | 8 | (26) |
|  | **FP Register** | | | | | | |
|  | Average | **Median (mins)** | |  | **Mean (mins)** | |  |
|  | Cells | Consultation | Recording | % | Consultation | Recording | % |
| Cambodia | 14 | 20 | 5 | (25) | 21 | 6 | (29) |
| Ghana | 12 | 15 | 3 | (20) | 15 | 5 | (33) |
| Mozambique | 66 | 20 | 10 | (50) | 21 | 15 | (71) |
| Nigeria | 17 | 10 | 2 | (20) | 9 | 3 | (33) |
| Tanzania | 18 | 30 | 5 | (17) | 31 | 5 | (16) |
|  | **EPI Register** | | | | | | |
|  | Average | **Median (mins)** | |  | **Mean (mins)** | |  |
|  | Cells | Consultation | Recording | % | Consultation | Recording | % |
| Cambodia | 10 | 15 | 5 | (33) | 16 | 6 | (38) |
| Ghana | 44 | 10 | 5 | (50) | 13 | 5 | (38) |
| Mozambique | 13 | 9 | 2 | (22) | 9 | 2 | (22) |
| Nigeria | 21 | 5 | 2 | (40) | 5 | 3 | (60) |
| Tanzania | 35 | 15 | 10 | (67) | 16 | 10 | (63) |

**Appendix Table 3b. Disease-specific registers– estimated consultation and recording time in five countries (2016-2017)**

| HIV-related | | | | | | | | |
| --- | --- | --- | --- | --- | --- | --- | --- | --- |
|  |  | Average | Median time | |  | Mean time | |  |
|  |  | Cells | Consultation | Recording | % | Consultation | Recording | % |
| Cambodia | HIV Testing for General Client Register | 14 | 20 | 5 | (25) | 20 | 6 | (30) |
|  | HIV Voluntary Testing Lab Register | 768 | 18 | 5 | (28) | 18 | 5 | (28) |
|  |  |  |  |  |  |  |  |  |
| Ghana | PMTCT Register | 11 | 13 | 4 | (31) | 19 | 8 | (42) |
|  |  |  |  |  |  |  |  |  |
| Mozambique | HIV CT Register Book | 19 | 15 | 10 | (67) | 23 | 10 | (43) |
|  | pre-ART register book | 14 | 18 | 4 | (22) | 18 | 4 | (22) |
|  | ART register book | 18 | 15 | 5 | (33) | 22 | 10 | (45) |
|  | PCR register book for infant early diagnosis | 14 | 10 | 1 | (10) | 10 | 1 | (10) |
|  | Viral load register book | 13 | 10 | 1 | (10) | 10 | 1 | (10) |
|  |  |  |  |  |  |  |  |  |
| Nigeria | ART register | 8 | 20 | 10 | (50) | 20 | 10 | (50) |
|  | HIV Counselling & Testing register | 14 | 5 | 2 | (40) | 12 | 1 | (8) |
|  | HCT Referral register | 16 | 2 | 2 | (100) | 2 | 2 | (100) |
|  | HIV Serology register | 14 | 15 | 2 | (13) | 18 | 2 | (11) |
|  | Treatment register | 10 | 2 | 2 | (100) | 2 | 2 | (100) |
|  | Daily HIV worksheet | 21 | 5 | 1 | (20) | 5 | 1 | (20) |
|  | HIV register | 13 | 10 | 2 | (20) | 10 | 2 | (20) |
|  | National PMTCT HTC Register | 19 | 10 | 2 | (20) | 16 | 2 | (13) |
|  | Child Follow up Register | 36 | 10 | 0 | (0) | 10 | 0 | (0) |
|  | PMTCT ARV Register | 16 | 13 | 2 | (15) | 13 | 2 | (15) |
|  | PMTCT Partner Register | 20 | 16 | 2 | (13) | 16 | 2 | (13) |
|  | PMTCT Dispensing worksheet | 11 | 10 | 4 | (40) | 10 | 4 | (40) |
|  |  |  |  |  |  |  |  |  |
| Tanzania | National HIV Care and Treatment ART Register | 22 | 27 | 9 | (33) | 40 | 11 | (28) |
|  | Pre-ART Register | 15 | 15 | 5 | (33) | 34 | 7 | (21) |
|  | HIV Voluntary Counselling and Testing Register | 17 | 30 | 3 | (10) | 28 | 4 | (14) |
|  | Asset Ledger (MSD) | 2 | 3 | 1 | (33) | 3 | 1 | (33) |
|  | Dispensing Register (MSD version) | 88 | 18 | 5 | (28) | 18 | 5 | (28) |
|  | ARVs Dispensing Register | 10 | 8 | 2 | (25) | 11 | 3 | (27) |
|  | Appointment Register | 6 | 5 | 3 | (60) | 7 | 3 | (43) |
|  | Lost-to follow-up HIV Clients Register | 6 | 3 | 1 | (33) | 3 | 1 | (33) |
|  | Follow-up after Initiation of ARV Guide | 11 | 15 | 3 | (20) | 14 | 3 | (21) |
|  | Weekly Assessment of ARV Adherence | 24 | 8 | 2 | (25) | 8 | 1 | (13) |
| TB-related | | | | | | | | |
|  |  | Average | Median time | |  | Mean time | |  |
|  |  | Cells | Consultation | Recording | % | Consultation | Recording | % |
| Cambodia | HC TB Patient register | 22 | 15 | 10 | (67) | 20 | 9 | (45) |
|  | HC TB Suspect Referral register | 17 | 20 | 7 | (35) | 18 | 8 | (44) |
|  | TB Lab Registration Form | 36 | 15 | 5 | (33) | 21 | 4 | (19) |
|  | TB Treatment Registration Form | 574 | 18 | 9 | (50) | 26 | 8 | (31) |
|  |  |  |  |  |  |  |  |  |
| Ghana | TB Quarterly Register | 39 | 30 | 4 | (13) | 30 | 4 |  |
|  |  |  |  |  |  |  |  |  |
| Mozambique | Isoniazide Prophylaxis for TB- register | 42 | 15 | 5 | (33) | 15 | 5 | (33) |
|  | TB cases register book | 17 | 20 | 8 | (40) | 22 | 8 | (36) |
|  | TB-MDR cases register book | 50 | 25 | 15 | (60) | 25 | 15 | (60) |
|  | TB smear tests & GeneXpert done by the lab | 19 | 5 | 3 | (60) | 5 | 3 | (60) |
|  |  |  |  |  |  |  |  |  |
| Nigeria | HF TB Central register | 20 | 18 | 2 | (11) | 19 | 2 | (11) |
|  | TB Clinic Suspect register | 16 | 13 | 2 | (15) | 13 | 2 | (15) |
|  |  |  |  |  |  |  |  |  |
| Tanzania | Collaborative TB/HIV Activities: TB Screening | 12 | 15 | 5 | (33) | 17 | 5 | (29) |

**Appendix Table 4a the number of consultations observed by service area in five countries (2016-2017)**

|  | Cambodia | Ghana | Mozambique** | Nigeria | Tanzania |
| --- | --- | --- | --- | --- | --- |
| OPD | 164* | 50 | 100 | 49 | 65 |
| ANC | 74 | 50 | 25 | 48 | 64 |
| FP | 60 | 50 | 25 | 34 | 65 |
| EPI | 80 | 50 | 26 | 50 | 80 |
| HIV | 0 | 0 | 25 | 24 | 65*** |
| TB | 1 | 0 | 26 | 10 |  |

* 74 were children <5 years who undergo IMCI assessment as part of OPD but NOT EPI

** In Mozambique 1 in 5 HIV consultations were for a 1st visit and the remaining were all follow-up visits; most of the TB consultations were for injection and prescription, or ART Refill

*** In Tanzania HIV visits involve also TB checks and controls

**Appendix Table 4b Comparing the mean consultation and register completion time (observed and self-reported) in five countries (2016-2017)**

|  |  | Consultation  (in mins) | | Recording  (in mins) | |
| --- | --- | --- | --- | --- | --- |
|  |  | Observed | Self-reported | Observed | Self-reported |
| OPD | Cambodia | 9 | 17 | 2 | 4 |
|  | Ghana | 10 | 18 | 3 | 5 |
|  | Mozambique | 6 | 13 | 2 | 5 |
|  | Nigeria | 7 | 10 | 2 | 2 |
|  | Tanzania | 8 | 10 | 4 | 5 |
| ANC | Cambodia | 12 | 28 | 4 | 5 |
|  | Ghana | 13 | 21 | 3 | 6 |
|  | Mozambique | 24 | 21 | 10 | 12 |
|  | Nigeria | 5 | 11 | 2 | 5 |
|  | Tanzania | 21 | 31 | 6 | 8 |
| FP | Cambodia | 11 | 21 | 3 | 6 |
|  | Ghana | 10 | 15 | 3 | 5 |
|  | Mozambique | 11 | 21 | 6 | 15 |
|  | Nigeria | 7 | 9 | 2 | 3 |
|  | Tanzania | 21 | 31 | 4 | 5 |
| EPI | Cambodia | 6 | 16 | 3 | 6 |
|  | Ghana | 12 | 13 | 5 | 5 |
|  | Mozambique | 4 | 9 | 2 | 2 |
|  | Nigeria | 4 | 5 | 2 | 3 |
|  | Tanzania | 8 | 16 | 4 | 10 |
| HIV_related | Cambodia | …. | 20 | …. | 6 |
|  | Ghana | …. | 19 | ….. | 8 |
|  | Mozambique* | 4 | 22 | 1 | 10 |
|  | Nigeria** | 5 | 12 | 1 | 1 |
|  | Tanzania*** | 13 | 18 | 3 | 5 |
| TB_related | Cambodia | 16 | 20 | …. | 9 |
|  | Ghana | …. | 30 | ….. | 4 |
|  | Mozambique+ | 5 | 15 | 2 | 5 |
|  | Nigeria++ | 3 | 19 | 1 | 2 |
|  | Tanzania+++ | 10 | 17 | 2 | 5 |

* ART register, observed mostly follow-up visits but first time visit could take up to 12 mins;

** HIV counselling and testing register;

***Dispensing Register (MSD version);

+ Isoniazide Prophilaxis for TB- register;

++ HF TB Central Register;

+++ Collaboartive TB/HIV Activties: TB Screening

**Appendix Table 5 – The Desk Review national inventory of reporting forms mandated and verified in use, 80 PHC facilities, five countries (2016-2017)**

| **Forms Grouping** | **Cambodia** | | | **Ghana** | | | **Mozambique** | | | **Nigeria** | | | **Tanzania** | | |
| --- | --- | --- | --- | --- | --- | --- | --- | --- | --- | --- | --- | --- | --- | --- | --- |
|  | **Counted** | **Year In** | **In use** | **Counted** | **Year In** | **In use** | **Counted** | **Year In** | **In use** | **Counted** | **Year In** | **In use** | **Counted** | **Year In** | **In use** |
| OPD | 1 | 1 | 1 | 2 | -- | 2 | 8 | 2 | 3 | 2 | 2 | 1 | 3 | 3 | 3 |
| RMNCAH | 1 | 1 | 1 | 8 | -- | 8 | 5 | -- | 4 | 4 | -- | 2 | 7 | 6 | 5 |
| Immunization | 2 | -- | 2 | 2 | -- | 2 | 3 | -- | 3 | 4 | 2 | 4 | 4 | -- | 3 |
| HIV_related | 3 | 3 | 3 | 2 | -- | 1 | 8 | -- | 5 | 3 |  | 2 | 10 | 3 | 9 |
| Malaria_related | 11 | 11 | 11 | 1 | -- | 1 |  |  |  |  |  |  |  |  |  |
| TB_related | 2 | 2 | 2 |  |  |  | 12 | 10 | 7 |  |  |  |  |  |  |
| Nutrition_related |  |  |  |  |  |  | 4 | 2 | 4 | 1 | -- |  | 4 | -- | 3 |
| Other* | 24 | 19 | 24 | 9 | -- | 9 | 12 | -- | 3 | 5 | 1 | 4 | 9 | 6 | 6 |
|  | 44 | 37 | 44 | 24 |  | 23 | 52 |  | 29 | 19 | 5 | 13 | 37 | 18 | 29 |

**Year In – Year introduced** is 2013/2014 or later; In Cambodia year of current version is 2015, only 6 reporting forms with unknown year ; Mozambique, 13 forms are under revision and 22 Unknown year; Nigeria, HIV/PMTCT are dated 2010 & 11 forms with year unknown ; Tanzania 19 reporting forms year unknown; Cambodia of the 44 forms , 28 are tally-sheets that were found in the facility but only 8 are in actual use.

**Appendix Table 6 The number of forms confirmed in use and the estimated reporting time (median) in monthly by service groupings in five countries (2016-2017)**

| Service grouping | Cambodia | | | Ghana | | | Mozambique+ | | | Nigeria | | | Tanzania | | |
| --- | --- | --- | --- | --- | --- | --- | --- | --- | --- | --- | --- | --- | --- | --- | --- |
|  | Form(s) | Median time (mins) | (%) | Form(s) | Median time (mins) | (%) | Form(s) | Median time (mins) | (%) | Form(s) | Median time (mins) | (%) | Form(s) | Median time (mins) | (%) |
| OPD | 1 | 1268 | (48) | 2 | 420 | (25) | 3 | 240 | (8) | 1 | 120 | (19) | 3 | 540 | (14) |
| RMNCAH | 1 | 30 | (1) | 9 | 582 | (34) | 3 | 480 | (17) | 2 | 35 | (6) | 5 | 620 | (16) |
| EPI | 1 | 60 | (2) | 1 | 63 | (4) | 2 | 720 | (25) | 4 | 72 | (11) | 3 | 225 | (6) |
| HIV | 1 | 25 | (1) | 1 | 38 | (2) | 3 | 340 | (12) | 2 | 195 | (31) | 9 | 881 | (22) |
| TB | 1 | 45 | (2) | 1 | 20 | (1) | 7 | 240 | (9) |  |  |  |  |  |  |
| Malaria | 5 | 568 | (21) | 1 | 50 | (3) |  |  |  |  |  |  |  |  |  |
| Other | 10 | 668 | (25) | 8 | 539 | (31) | 7 | 960 | (34) | 4 | 205 | (33) | 9 | 1660 | (42) |
| Total | 20 | 2664 |  | 23 | 1712 |  | 25 | 2850* |  | 13 | 627 |  | 29 | 3926 |  |
| (In hours) |  | 44 |  |  | 29 |  |  | 48 |  |  | 10 |  |  | 65 |  |

+ Includes 2 IPD reporting forms which require about 480 mins per month.

**Appendix Table 7 – Distribution of reporting forms (cells and estimated time), by service area, in five countries (2016-2017)**

| Cambodia (N=20) | |  | | | Estimated time | |
| --- | --- | --- | --- | --- | --- | --- |
| Grouping | **Report name** | **Mandatory (PHC)** | **Due to disaggregation** | **Averagely entered** | **MEDIAN** | **MEAN** |
| OPD | HC Monthly Report (HC1 Form) | 4632 | **4403** | 732 | 1268 | 1105 |
| MNCH | HC Monthly Report on Infant and Child Vaccination | 66 | 0 | 142 | 30 | 125 |
| EPI | HC Monthly EPI Report | 100 | 0 | 106 | 60 | 65 |
| NCD | Cervical Cancer Screening Test (VIA) Monthly | 13 | 0 | 13 | 33 | 33 |
| HIV | Monthly Report for PMTCT/HIV Testing and | 70 | 0 | 72 | 25 | 44 |
| TB | TB Monthly Report | 233 | 22 | 161 | 45 | 158 |
| Malaria | HC Malaria Tally Sheet | 42 | 0 | 22 | 40 | 40 |
|  | HC Monthly Bednet Distribution Report | 180 | 0 | 198 | 45 | 43 |
|  | OD Malaria Investigation and Response Form | 74 | 0 | 150 | 120 | 120 |
|  | Monthly Report on Malaria Education form | 128 | 0 | 128 | 60 | 60 |
|  | HC Monthly Report on Malaria Active Case | 60 | 0 | 70 | 20 | 20 |
| STD | HC STD Monthly Report | 74 |  | 66 | 15 | 15 |
| Finance | Health Equity Fund Report | 54 | 0 | 49 | 30 | 50 |
|  | HC Quarterly Health Financing Report | 1216 | 0 | 140 | 240 | 249 |
| NGO | Voucher Program Report (for Action for H | 123 | 0 | 97 | 20 | 58 |
|  | Community-Based Health Insurance Report | 40 | 0 | 27 | 18 | 24 |
| Pharma | Drugs and Consumables Consumption Report | 91 | 0 | 43 | 90 | 209 |
|  | Stock/Bin Card (for HC Pharmacy) | 216 | 0 | 149 | 360 | 609 |
|  | HC Drugs & Consumables Consumption Report | 540 | 0 | 242 | 120 | 393 |
| Staff | HC Special Operating Agency (SOA) Performance | 23 | 0 | 21 | 25 | 27 |
|  | (In mins) |  |  |  | 2664 | 3447 |
|  | (In hours) |  |  |  | 44 | 57 |
| Ghana (N=23) | |  | | | **Estimated time** | |
| Grouping | **Report name** | **Mandatory (PHC)** | **Due to disaggregation** | **Averagely entered** | **MEDIAN** | **MEAN** |
| OPD | Monthly OPD Morbidity Returns | 2350 | 2243 | 2350 | 270 | 445 |
|  | Statement of Outpatient | 135 | 130 | 135 | 150 | 267 |
| MNCH | Form A-Monthly Midwifes Returns | 200 | 42 | 200 | 120 | 138 |
|  | Form B-Family Planning Returns | 350 | 6 | 350 | 63 | 205 |
|  | Form C-Monthly Child Health Returns | 114 | 65 | 114 | 48 | 91 |
|  | Form D-Monthly RCH Returns | 59 | 0 | 59 | 8 | 8 |
|  | Yaws Monthly Report | 58 | 2 | 58 | 90 | 90 |
|  | CBA Monthly Reporting Form | 27 | 0 | 23 | 20 | 48 |
|  | Family Planning monthly summary | 400 | 0 | 400 | 165 | 165 |
|  | Monthly Nutrition Report | . | . | 204 | 30 | 84 |
|  | PNC Report | 25 | . | 25 | 38 | 41 |
| EPI | Monthly Vaccination Report | 321 | 108 | 321 | 63 | 304 |
| Lab | Medical Lab Report | 2322 | . | 2322 | 240 | 240 |
| HIV | PMTCT Monthly Report | 158 | 130 | 158 | 38 | 41 |
| Malaria | Monthly Malaria Data Returns on Anti-Malarial | 68 | 24 | 68 | 50 | 40 |
| IDSR | IDSR Weekly | 94 | 8 | 94 | 25 | 22 |
|  | IDSR Monthly | 190 | 16 | 190 | 20 | 27 |
| Pharma | Bin Card | 13 | 0 | 13 | 5 | 5 |
|  | Store Ledger | 211 | 0 | 11 | 180 | 180 |
|  | Way Bill | 20 | 0 | 5 | 2 | 2 |
|  | Inventory Control Card | 12 | 0 | 15 | 2 | 3 |
| Others | NHIA Claim Form | 38 | 1 | 38 | 5 | 7 |
|  | TB Quarterly Report | . | . | 799 | 20 | 20 |
|  | (In mins) |  |  |  | 1712 | 2533 |
|  | (In hours) |  |  |  | 29 | 42 |
| Mozambique (N=25) | |  | | | **Estimated time** | |
| Grouping | **Report name** | **Mandatory (PHC)** | **Due to disaggregation** | **Averagely entered** | **MEDIAN** | **MEAN** |
| OPD | OPD summary sheet | 12 | 6 | 12 | 240 | 340 |
|  | Stomatology summary sheet | 3 | 0 | 6 | 80 | 80 |
|  | RDT and AL consumption form | 14 | 0 | **20** | 30 | 30 |
| IPD | Maternity ward summary sheet | 83 |  | 28 | 180 | 195 |
|  | Gynaecology emergency summary sheet | 51 |  | 59 | 300 | 300 |
| RMNCH | ANC summary sheet | 43 | 4 | 31 | 480 | 550 |
|  | Family Planning summary sheet | 66 | 4 | 41 | 720 | 630 |
|  | PNC summary sheet | 47 | 0 | 0 | 1200 | 1200 |
| EPI | EPI summary sheet | 136 | 136 | 51 | 420 | 420 |
|  | Summary sheet for: Administration of Vit. A and Mebendazole (6-11 months) | 37 | 15 | **1** | 620 | 620 |
| Nutrition | Acute malnutrition treatment in OPD summary sheet | 73 | 73 | 6 | 330 | 470 |
|  | Acute malnutrition intra-hospital treatment (0-14 years) summary sheet | 36 | 36 | 30 | 120 | 120 |
|  | Consumption form of therapeutic products for acute malnutrition | 30 | 0 | 0 | 240 | 240 |
|  | Child nutrition surveillance summary sheet | 6 | 2 | 1 | 480 | 480 |
| HIV/PTMCT | Child care for children at risk | 47 | 0 | 20 | 480 | 576 |
|  | HIV CT summary sheet | 373 | 10 | 0 | 60 | 60 |
|  | ARV treatment summary sheet | 126 | 126 | 52 | 480 | 408 |
| TB | Anti-Tetanus immunization in pregnant women summary sheet | 21 | 0 | 20 | 960 | 960 |
|  | Quarterly notification form of TB cases (new and retreatment) | 57 | 18 | 4 | 100 | 300 |
|  | Quarterly evaluation form of treatment results for TB cases (new BK+) | 119 | 119 | 0 | 320 | 960 |
|  | Quarterly form TB and MDR cases | 19 | 0 | 20 | 160 | 480 |
|  | Quarterly summary sheet for MDR-TB treatment results | 18 | 0 | 12 | 240 | 720 |
|  | NTB summary sheet | 19 | 0 | 10 | 135 | 135 |
|  | TB-MDR cases form | 11 | 0 | 18 | 360 | 360 |
| STI | STIs summary sheet | 22 | 12 | 10 | 570 | 570 |
|  | (In mins) |  |  |  | 9305 | 11204 |
|  | (In hours) |  |  |  | 155 | 187 |
| Nigeria (N=13) | |  | | | **Estimated time** | |
| Grouping | **Report name** | **Mandatory (PHC)** | **Due to disaggregation** | **Averagely entered** | **MEDIAN** | **MEAN** |
| OPD | NHMIS Monthly Summary Form | 585 | 319 | 585 | 120 | 143 |
| EPI | Facility monthly Immunization summary | 64 | 34 | 109 | 30 | 64 |
|  | Vaccines Utilization Summary Form | 205 | 0 | 205 | 20 | 72 |
|  | Line list form for immunization | . | . | 20 | 15 | 15 |
|  | Vaccine/ diluents/injection equipment request form | 33 | 0 | 23 | 7 | 7 |
| RMNCH | Family planning monthly integration summary | 13 | 4 | 13 | 5 | 5 |
|  | 4HF-2 monthly report of family planning | . | 0 | 0 | 30 | 30 |
| HIV/PTMCT | PMTCT Monthly Summary Form | 103 | 34 | 105 | 75 | 75 |
|  | HCT Monthly Summary Form | 306 | 108 | 306 | 120 | 110 |
| Pharma | Requisition Issue and report form SDP | 20 | . | 41 | 15 | 15 |
|  | Daily consumption record | 11 | 0 | 11 | 20 | 20 |
|  | Combined reports requisition issue & rec | 16 | . | 32 | 130 | 130 |
| Disease notification | Routine monthly notification form | 908 | 748 | **1113** | 40 | 40 |
|  | (In mins) |  |  |  | 627 | 726 |
|  | (In hours) |  |  |  | 10 | 12 |

| Tanzania (N=29) | |  | | | Estimated time | |
| --- | --- | --- | --- | --- | --- | --- |
| Grouping | **Report name** | **Mandatory (PHC)** | **Due to disaggregation** | **Averagely entered** | **MEDIAN** | **MEAN** |
| OPD | OPD Report Form | 1386 | 648 | 1386 | 360 | 422 |
|  | DTC Report Form | 90 | 36 | 90 | 60 | 77 |
|  | Malaria Laboratory Testing Report Form | 55 | 15 | 18 | 120 | 147 |
| IPD | IPD Report Form | 1088 | 355 | 1088 | 600 | 550 |
| RMNCH | ANC Report Form | 150 | 50 | 156 | 150 | 275 |
|  | Child Report Form | 252 | 84 | 252 | 180 | 372 |
|  | Monthly Family Planning Report Form | 318 | 62 | 318 | 120 | 271 |
|  | Labour & Delivery Report Form | 219 | 73 | 219 | 90 | 143 |
|  | PNC Report Form | 120 | 40 | 120 | 80 | 101 |
| EPI | Monthly Health Facility report on IVD activities | 290 | 0 | 280 | 120 | 120 |
|  | Monthly Report on Consumption of Reagents | 8 | 0 | 8 | 15 | 15 |
|  | Form of Consumption and Quality Assurance | 8 | 0 | 8 | 90 | 103 |
| STI | Facility Monthly STI Summary Form | 164 | 39 | 164 | 35 | 77 |
| HIV/PTMCT | Tracer Medicine Report Form | 90 | 0 | 83 | 8 | 8 |
|  | PMTCT Quarter Report Form | 19 | 0 | 20 | 30 | 54 |
|  | Quarterly facility-based HIV Care/ART Report | 195 | 57 | 175 | 90 | 154 |
|  | Cohort Analysis Reporting Form | 136 | 0 | 136 | 240 | 280 |
|  | HIV Counseling & Testing Report | 114 | 24 | 114 | 120 | 97 |
|  | Monthly Report on Consumption of ARVs & OIs | . | . | 6 | 18 | 24 |
|  | Dry Blood Specimen (DBS) Form | 29 | 0 | 29 | 10 | 10 |
|  | Specimen Collection Log | 7 | 0 | 7 | 5 | 5 |
|  | Elizabeth Glacer Paediatric AIDS Foundation Community Linkage Report | 95 | 0 | 95 | 360 | 360 |
| Pharma/Supplies | Request and Reporting of Medicine and Medical Devices (MDS) | 35 | 0 | 8 | 15 | 15 |
| Nutrition | Home-based Care Monthly Report Form | 68 | 2 | 68 | 60 | 55 |
|  | Monthly Summary Form for NACS Service | 279 | 42 | 279 | 225 | 225 |
|  | Malnutrition Treatment Monthly Report F | 19 | 0 | 19 | 40 | 40 |
| Others | Dental Report Form | 513 | 171 | 432 | 180 | 180 |
|  | Gender-based Violence Report Form | 313 | 48 | 313 | 25 | 25 |
|  | Report and Request Form for PHC | . | . | 400 | 480 | 480 |
|  | (In mins) |  |  |  | 3926 | 4685 |
|  | (In hours) |  |  |  | 65 | 78 |
